# A Visual Remote Associates Test and Its Validation

**DOI:** 10.3389/fpsyg.2020.00026

**Published:** 2020-01-28

**Authors:** Ana-Maria Olteţeanu, Faheem Hassan Zunjani

**Affiliations:** Cognitive Systems Group, Human-Centered Computing Lab, Freie Univeristät Berlin, Berlin, Germany

**Keywords:** visual, remote associates test, creativity tests, normative data, human creativity, computational creativity

## Abstract

The Remote Associates Test (RAT) is a widely used test for measuring creativity, specifically the ability to make associations. The Remote Associates Test normally takes a linguistic form: given three words, the participant is asked to come up with a fourth word associated with all three of them. While visual creativity tests do exist, no creativity test to date can be given in both a visual and linguistic form. Such a test would allow the study of differences between various modalities, in the context of the same creative process. In this paper, a visual version of the well-known Remote Associates Test is constructed. This visual RAT is validated in relation to its linguistic counterpart.

## 1. Introduction

Humans are capable of creativity across a wide variety of tasks and domains, including the linguistic (e.g., solving riddles, writing novels), visual (e.g., visual arts, design, object-related creativity), auditory (e.g., musical), tactile (e.g., fashion and fabrics, texture), gustatory, and olfactive (e.g., culinary creativity, perfume), etc. Creativity in many domains runs across various sensory or linguistic modalities (e.g., literature, scientific discovery, innovation).

Complex creativity tasks, like the solving of practical insight problems, might elicit both linguistic and visual creativity. Creativity batteries of tests which include both visual and linguistics tests do exist—like the Torrance Tests of Creative Thinking (TTCT), which contains both verbal and figural tests (Kim, 2006). However, no creativity evaluation task or test exists which can be given separately in both linguistic and visual forms, thus affording cross-domain comparison of a particular set of creative processes. The usefulness of such a test would be to: (i) check whether the same creative processes act across the visual and linguistic domain; (ii) compare performance results in various domain; and (iii) posit domain-relevant differences.

Aiming to fill this gap, this paper takes a well established creativity test, the Remote Associates Test (Mednick and Mednick, 1971) and describes an approach toward developing a visual derivate of this test.

A computational linguistic solver for this test—comRAT-C (Olteţeanu and Falomir, 2015) was previously implemented under a theoretical creative problem-solving framework (CreaCogs; Olteţeanu, 2014; Olteţeanu, 2016). Part of the formalization for comRAT-C is used to inform the creation of a visual form of the Remote Associates Test.

The rest of the paper is organized as follows. The Remote Associates Test and the construction of its visual counterpart (vRAT) are discussed in the next section. Two studies with human participants who were given vRAT queries are described in the Studies section. Results of these studies are presented in results section. A discussion on the visual RAT items and normative data takes place after the results section, where further work is also proposed.

## 2. An Approach for Creating the Visual Remote Associates Test

The Remote Associates Test (RAT), originally devised by Mednick and Mednick (Mednick and Mednick, 1971), aims to reflect the creative ability of the participant through measuring their skill at remote compound linguistic association. In the RAT, participants are given three words—like cream, skate and water—and asked to come up with a fourth word which relates to all of them. A good answer to this particular query is ice.

The Remote Associates Test has been widely used in the literature (Dorfman et al., 1996; Ansburg, 2000; Ansburg and Hill, 2003; Ward et al., 2008; Cai et al., 2009; Cunningham et al., 2009). Stimuli for this test exist not just in English (Bowden and Jung-Beeman, 2003; Olteţeanu et al., 2017), but also German (Landmann et al., 2014), Chinese (Shen et al., 2016; Wu and Chen, 2017), Italian (Salvi et al., 2016), Romanian (Olteţeanu et al., 2019b), etc. An approach toward generating functional RAT queries has also been proposed (Olteţeanu et al., 2019a), enhancing the repository of available RAT queries. Furthermore, a computational solver exists that solves the compound RAT (Olteţeanu and Falomir, 2015) and correlates in performance (both Accuracy and Response Times) with existing normative data (Bowden and Jung-Beeman, 2003). Also, a computational generator of RAT queries was implemented (Olteţeanu et al., 2017) and shown to be useful in designing empirical explorations with a high degree of control (Olteţeanu and Schultheis, 2017).

Bowden and Jung-Beeman have proposed normative data on 144 compound RAT problems (Bowden and Jung-Beeman, 2003). Besides the compound (or structural) form of the Remote Associates Test, in which the relationship between query words and answer words is linguistic, Worthen and Clark (Worthen and Clark, 1971) argued that some of the items proposed by Mednick and Mednick are functional—that is the relationship between them (e.g., items like “bird” and “egg”), rather than just a structural one (e.g., items like “black” and “magic”). The functional items proposed by Worthen and Clark were lost, however a computational implementation aimed to recover their concept by generating functional versions of the RAT (Olteţeanu et al., 2019a).

In a previous formalization which aimed at computationally solving this test (Olteţeanu and Falomir, 2015), the Remote Associates Test was described as follows: three words *w*_*a*_, *w*_*b*_, *w*_*c*_ are given to the participant; a word which relates to all these words needs to be found, *w*_*x*_. In solving the compound RAT, (*w*_*a*_, *w*_*x*_), (*w*_*b*_, *w*_*x*_), and (*w*_*c*_, *w*_*x*_) or the reverse ordered terms (*w*_*x*_, *w*_*a*_), (*w*_*x*_, *w*_*b*_), (*w*_*x*_, *w*_*c*_) have to be composed words in the language in which the RAT is given in. In the case of composed words, *w*_*z*_ might be a word composed of a query word and a solution word, (*w*_*x*_*w*_*a*_) or (*w*_*a*_*w*_*x*_). For example, for the query aid, rubber and wagon, the answer term band constructs joint composed words with some of the query terms (band-aid, bandwagon), but not with others (rubber band). Note that the answer word is also not in the same position in the three linguistic structures.

In order to devise a visual RAT, the following approach extends this formalization from the linguistic to the visual domain. Thus, if the terms *w*_*a*_, *w*_*b*_, *w*_*c*_, and *w*_*x*_ stood for words in the linguistic RAT, in this visual approach they stand for visual representations of objects and scenes. The visual RAT can be described thus as follows: Given visual entities *w*_*a*_, *w*_*b*_, *w*_*c*_, there exists an entity *w*_*x*_, which generally co-occurs visually with the other shown entities *w*_*a*_, *w*_*b*_ and *w*_*c*_.

**Figure 1:**
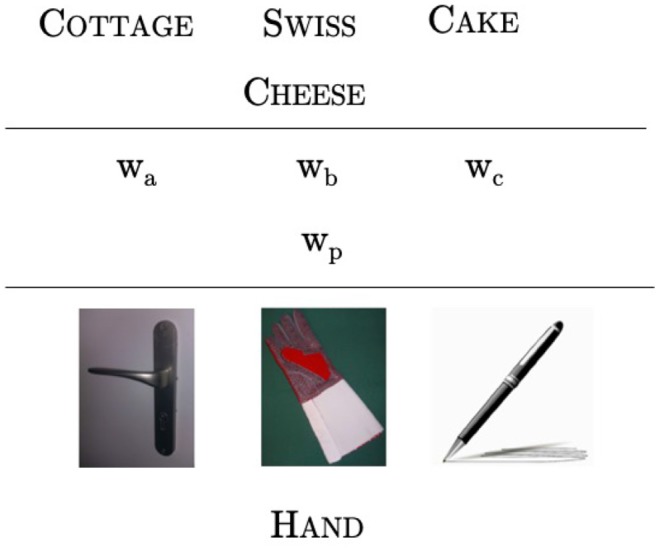
Visual representation of the process of creation of vRAT queries. A linguistic query was formalized and then turned into a visual query. This particular vRAT query is the first training query.

Applying this approach, visual queries can be created. For example, Figure 1 provides visual representations of the objects glove, handle and pen. An appropriate answer to this query is hand, because hands are visual entities that co-occur with each of the given three objects.

The visual entity hand can be considered a *visual associate* of each of the initial objects glove, handle and pen. This notion of a *visual associate* is intended to play in this approach a role analogous to that of a linguistic associate in the linguistic RAT. If compound associates are possible in the linguistic compound RAT (*band* and *aid*) and functional associates are possible in the linguistic functional RAT (*bird* and *egg*) (Olteţeanu et al., 2019a), visual associates are meant to encompass both categories. A further differentiation could be made between objects that co-occur together visually (compound-like) and objects that afford interactions between them (functional-like). This work will focus on establishing the visual RAT, without delving deeper into this differentiation. Each initial object is thus considered to have a variety of visual associates. Visual associate pairs which co-occur together, in a previously encountered visual scene or experience, play the role that composed words or linguistic structures in which *w*_*a*_ and *w*_*x*_ co-occur. Thus, for a natural or artificial cognitive system to be able to solve queries like the one in Figure 1, it needs to be acquainted with visual experiences containing the visual entities in it (hand, glove), (handle, hand), and (pen, hand).

**Figure 2:**
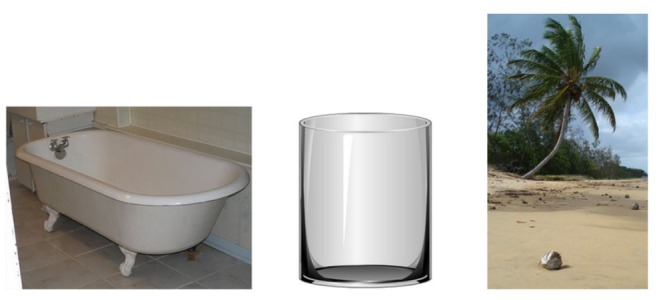
A visual RAT item showing participants a bathtub, glass, and beach.

Visual queries do not have to involve body parts of the solver that interact with the given object —they could also represent objects in the environment and scenes. For example, Figure 2 shows participants a bathtub, glass and beach. A visual item co-occurring with each of them and thus a potential answer is water. The visual representations were chosen or crafted so that they do not show the answer—thus an empty bathtub and empty glass are presented, and only a part of a beach that does not depict the sea is displayed.

This approach can be summarized as follows:

- Visual objects or scenes replace words and expressions;
- Visual relationships between objects take the place of linguistic relationships—be it relationships of co-occurrence or functional;
- The solver is expected to rely on principles of visual association more often than on linguistic association ones.

Using this approach, a set of visual RAT queries was manually created. In the following section, this test is evaluated in comparison to the linguistic RAT, validated and considered in relation to other measures.

## 3. Studies

In order to evaluate the performance of humans in the visual RAT items designed, to construct an initial set of normative data and assess the potential relationships between performance in the visual RAT and performance in the linguistic compound RAT, two studies were conducted. The first study was completed by 42 participants that have previously solved the compound RAT. In order to validate the results, a second study was set up, for which power was calculated based on the result of the first study. In the second study, participants solved both visual and linguistic queries, and measures of verbal fluency were also applied.

### 3.1. Study 1

#### 3.1.1. Method

Using the approach described in the previous section, a set of 46 visual RAT queries was manually created. These queries were administered to the participants of the study for validation.

#### 3.1.2. Procedure

Participants for Study 1 were recruited using Figure-Eight (F8)^1^, and then directed to our own website, were the study was set up using jsPsych^2^. In the survey created for this study, the participants were first asked about their age bracket, sex and education level. Age brackets provided were: “Under 20 years old,” “20–30 years old,” “30–40 years old,” “40–50 years old,” “50–60 years old,” “60–70 years old,” “Over 70 years old”); sex : “Male,” “Female”; education level: “Secondary school,” “High school diploma,” “Enrolled in undergraduate courses (University),” “Completed under-graduate course/Graduated,” “Enrolled in postgraduate courses (University),” “Completed postgraduate course.”

After this, participants were asked to provide a self-assessment of their creativity, and problem-solving skill (“Low,” “Average,” “Above-Average,” “High,” “Very High”).

The participants were then provided instructions with example vRAT queries. They were then presented with 46 vRAT queries in randomized order.

#### 3.1.3. Participants

**Figure 3:**
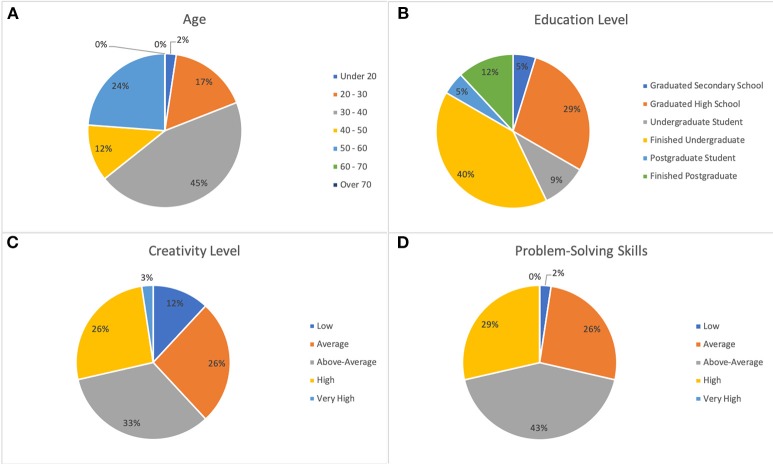
Demographic distribution of Study-1 participants. **(A)** Age. **(B)** Education level. **(C)** Creativity level. **(D)** Problem-solving skills.

The participants from a previous study on compound RAT queries created with a computational solver comRAT-G (Olteţeanu et al., 2017) were invited to solve the visual RAT queries. Of the previous compound RAT queries solvers, 42 people (28 females and 14 males) participated; of these, four participants left more than 20% queries of either visual or linguistic RAT unattempted and hence the data analysis has been done for N = 38. Figure 3 shows the demographic distribution of the Study-1 participants. The majority of the participants belonged to age bracket of 30-40 years (19), had finished their undergraduate degree (17) and had rated their creativity (14) and problem-solving skills (18) as “above-average.”

#### 3.1.4. Creativity Metrics

Accuracy was a creativity metric used for this study: *vRAT accuracy, comRAT-G accuracy, B-JB accuracy* and *linguistic RAT accuracy*. These stood for:

– *vRAT accuracy*: the number of correctly answered vRAT queries'
– *comRAT-G accuracy*: the number of correctly answered queries from the corpus created with comRAT-G (Olteţeanu et al., 2017) and
– *B-JB accuracy*: the number of correctly answered Bowden & Jung-Beeman queries.

The *comRAT-G accuracy* and *B-JB accuracy* were taken from the participants' previous performance in the comRAT-G study. This was compared to their performance in the visual RAT. The *linguistic RAT accuracy* is the sum of *comRAT-G accuracy* and *B-JB accuracy*, denoting the total number of linguistic RAT queries answered correctly.

Response Times were the second metric used. *vRAT RT, comRAT-G RT* and *B-JB RT* recorded the mean response times of the participants when correctly answering the corresponding vRAT, comRAT-G, and B-JB queries.

### 3.2. Study 2

#### 3.2.1. Method

For further validation, the same set of vRAT queries used in the first study was also used in the second study. To study the potential relationships between performance in the linguistic RAT and the visual RAT, participants were presented with 48 visual RAT queries to solve, and then with 48 linguistic RAT queries. The linguistic RAT queries were a randomized mix of 24 comRAT-G queries (Olteţeanu et al., 2017) and 24 queries from the Bowden & Jung-Beeman dataset (Bowden and Jung-Beeman, 2003). The 48 queries were the same for all participants but were presented to each of them in a different order.

#### 3.2.2. Procedure

The participants for the second study were recruited using two platforms: Figure-Eight (F8) and Mechanical Turk (MTurk)^3^. After enrolling for the test on either of the platforms, they were redirected to our website where the study was setup using jsPsych.

Each participant was first asked a set of questions about their gender, age group, education, creativity and problem-solving skills. After the demographic questions, the participants were administered two verbal fluency tests: a phonemic test (with “F,” “A,” “S” as stimuli letters) and a semantic test (with “animal,” “fruit” and “furniture” as categories). In these verbal fluency tests, participants were asked to list as many words as they could in one minute for each of the verbal fluency stimuli.

Then, the participants were presented with the instructions for solving the visual RAT, two example queries and their answers. After this, they were presented with visual RAT queries in random order. The response time for every response was recorded.

After solving visual RAT queries, participants were presented with the instructions for solving the linguistic RAT followed by two example queries one by one and asked to try to answer them before the correct answers were revealed to them. After this, the participants were asked to solve 48 linguistic RAT queries.

#### 3.2.3. Participants

**Figure 4:**
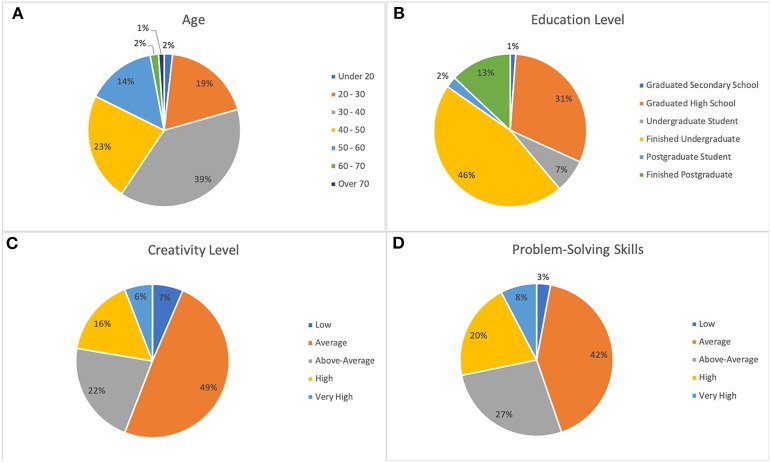
Demographic distribution of Study-2 participants. **(A)**: Age. **(B)**: Education level. **(C)**: Creativity level. **(D)**: Problem-solving skills.

26 people (15 female and 11 male) participated from F8 and 144 people (67 female and 77 male) participated from MTurk. Figure 4 shows the demographic distribution of the 170 participants in Study-2. The majority of participants belonged to the age bracket of 30–40 years (66), had finished an undergraduate degree (78) and had rated their creativity (84) and problem-solving skills (71) as “average.” 3 participants from F8 and 6 participants from MTurk had left more than 20% of the queries unanswered and hence were not included in the data analysis.

#### 3.2.4. Creativity Metrics

F-A-S Test (Patterson, 2011) is a verbal fluency test where a participant lists as many words that they can think of starting with the letters “F,” “A,” and “S” within a specified timeframe, usually 1 min for each of those letters. The *F score, A score* and *S score* recorded the number of words participants produced starting with the corresponding letters. *FAS score* was calculated as the sum of these three scores. The *Category score* recorded the number of words listed by the participant for the three categories. The accuracy metrics and their corresponding response time metrics were recorded in the same way as in Study-1.

## 4. Results

The following section presents the results of the two studies.

### 4.1. Results - Study-1

#### 4.1.1. Descriptive Data

Each vRAT query was answered correctly on average by 18.8 participants (*SD* = 11.98). The query “BOTTLE-GRAPE-CELLER”(answer: “WINE”) was found to be the easiest query with 40 participants answering it correctly. “HAND MIRROR-PURSE-RED MARK” (answer: “LIPSTICK”) was the most difficult query to answer with only 1 participant answering it correctly. 21 (45.65%) vRAT queries out of a total of 46 were answered by more than half of the participants.

#### 4.1.2. Correlations

The correlation between *vRAT accuracy* and *comRAT-G accuracy* was observed to be 0.431 (*p* < 0.01), as shown in Table 1. For calculating the correlations between response times, outliers were found using the Inter-Quartile Range method and removed. A significant correlation between response times of correct responses for vRAT and linguistic RAT queries was observed (*n* = 38, *r* = 0.477, *p* < 0.002).

**Table 1:** Correlations of the linguistic RAT metrics with the vRAT metrics.

	**vRAT (*****N*** **= 38)**			
	**Accuracy**		**RT correct**	
	***r*-value**	***p*-value**	***r*-value**	***p*-value**
comRAT-G	0.431^**^	0.007	0.432^**^	0.007
B-JB	0.022	0.894	0.444^**^	0.005
Linguistic RAT	0.202	0.224	0.477^**^	0.002

Correlations significance level indicated as follows: 0.05 — “*”; 0.01 — “

** *”*.

#### 4.1.3. Validity

As a reliability metric, Cronbach's alpha was calculated for the vRAT data gathered. Cronbach's alpha is a measure of internal consistency, that is, how closely related a set of items are as a group. It is considered to be a measure of scale reliability. A Cronbach's alpha above 0.75 is considered to show a high internal validity. An alpha value of 0.751 was observed for the accuracy of the participants in vRAT queries.

### 4.2. Results - Study-2

The responses of all the participants from both the platforms was put into three samples: data from F8 participants, data from MTurk participants and combined data from all the F8 and MTurk participants for the analysis.

#### 4.2.1. Descriptive Data

Each vRAT query was solved on an average by 15.62 participants (*SD* = 6.01). Each participant on an average spent 13.0 s (*SD* = 7.22) on the vRAT queries. For the total phonemic verbal fluency metric (*FAS score*), a mean of 46.96 words (*SD* = 11.71) was observed. For the total semantic verbal fluency metric (*Category score*), a mean of 41.64 words (*SD* = 11.33) was observed. Table 2 shows detailed statistics on the verbal fluency scores and RAT accuracy metrics. Table 3 shows the descriptive statistics on response time metrics.

**Table 2:** Means and Standard Deviations of verbal fluency scores and accuracy performance on the visual and linguistic Remote Associates Test–Study-2.

**Metric**	**Figure-Eight**	**Mechanical Turk**	**Combined F8-MT**
	***N* = 26**	***N* = 144**	***N* = 170**
	**Mean (*SD*)**	**Mean (*SD*)**	**Mean (*SD*)**
F score	15.17 (4.03)	16.33 (4.09)	16.16 (4.09)
A score	13.17 (4.02)	13.93 (4.03)	13.83 (4.02)
S score	15.13 (5.33)	17.28 (4.68)	16.97 (4.82)
FAS score	43.48 (12.62)	47.54 (11.50)	46.96 (11.71)
Category score	38.09 (12.01)	42.23 (11.15)	41.64 (11.33)
vRAT accuracy	15.17 (5.99)	15.70 (6.03)	15.62 (6.01)
comRAT-G accuracy	8.65 (4.81)	9.29 (2.91)	9.20 (3.24)
B-JB accuracy	10.91 (7.39)	11.62 (5.32)	11.52 (5.64)
Linguistic RAT accuracy	19.57 (10.89)	20.91 (6.77)	20.71 (7.47)

**Table 3:** Means and Standard Deviations of response times for the visual and linguistic Remote Associates Tests – Study-2.

**Response Times**	**Figure-Eight**	**Mechanical Turk**	**Combined F8-MT**
	***N* = 26**	***N* = 144**	***N* = 170**
	**Mean (*SD*)**	**Mean (*SD*)**	**Mean (*SD*)**
All vRAT	15.20 (8.37)	12.64 (6.98)	13.00 (7.22)
Correct vRAT	11.38 (5.54)	10.65 (11.65)	10.75 (10.98)
All comRAT-G	27.35 (31.89)	15.47 (10.16)	17.17 (15.67)
Correct comRAT-G	16.42 (12.74)	10.49 (8.19)	11.34 (9.17)
All B-JB	21.82 (14.94)	13.33 (7.65)	14.54 (9.47)
Correct B-JB	14.57 (9.57)	9.53 (5.83)	10.25 (6.70)
All linguistic RAT	24.59 (19.11)	14.40 (8.19)	15.86 (10.97)
Correct linguistic RAT	15.12 (9.03)	9.97 (6.22)	10.70 (6.90)
All vRAT & linguistic RAT	19.99 (13.66)	13.54 (6.90)	14.46 (8.46)
Correct vRAT & linguistic RAT	13.28 (6.50)	10.32 (6.46)	10.74 (6.53)

#### 4.2.2. Correlations

Significant correlations of *vRAT accuracy* with all the linguistic RAT accuracy metrics were observed [*linguistic RAT*: (*n* = 170, *r* = 0.30, *p* < 0.001)], as shown in Table 4. High significant correlations were also observed between the phonemic and semantic verbal fluency metrics (*n* = 170, *r* = 0.65, *p* < 0.001).

**Table 4:** Correlations between all scoring and accuracy metrics for all the participants of Study-2.

	**F**	**A**	**S**	**FAS**	**Category**	**vRAT**	**comRAT-G**	**B-JB**	**Linguistic RAT**
F	-	0.70^***^	0.72^***^	–	0.54^***^	0.03	0.25^**^	0.18^*^	0.25^**^
A		–	0.76^***^	-	0.58^***^	0.03	0.29^***^	0.15	0.24^**^
S			–	–	0.64^***^	0.04	0.28^***^	0.23^**^	0.30^***^
FAS				–	0.65^***^	0.04	0.30^***^	0.21^**^	0.29^***^
Category					–	0.004	0.29^^***^^	0.26^^***^^	0.33^^***^^
vRAT						–	0.33^^***^^	0.21^**^	0.30^^***^^
comRAT-G							–	0.37^^***^^	–
B-JB								–	–
Linguistic RAT									–

Correlations significance level indicated as follows: 0.05 – “

* ”; 0.01 – “

** ”; 0.001 – “

*** *” N = 170*.

Significant correlations were also observed between verbal fluency of both phonemic and semantic types with the linguistic RAT, on both comRAT-G produced [*FAS*: (*n* = 170, *r* = 0.30, *p* < 0.001), *Category*: (*n* = 170, *r* = 0.29, *p* < 0.001)] and B-JB stimuli [*FAS*: (*n* = 170, *r* = 0.21, *p* < 0.01), *Category*: (*n* = 170, *r* = 0.26, *p* < 0.001)] datasets. A significant correlation of 0.37 was also observed between the performance of participants in comRAT-G queries and Bowden & Jung-Beeman queries.

For calculating the correlations between response times, outliers were found using the Inter-Quartile Range method and removed. High significant correlations were observed between the response times for vRAT and linguistic RAT queries (*n* = 170, *r* = 0.70, *p* < 0.001); this was a consequence of correlations between performance in the visual RAT and the comRAT-G items, and also between the visual RAT and B-JB items. A high significant correlation was also observed between the response times for the correct responses for comRAT-G queries and the Bowden & Jung-Beeman queries (*n* = 170, *r* = 0.49, *p* < 0.001). Table 5 shows the correlations between the response times for the RAT metrics.

**Table 5:** Correlations of response times for all metrics for all the participants of Study-2.

	**vRAT**	**vRAT correct**	**B-JB**	**B-JB correct**
comRAT-G	0.64^***^	–	0.49^***^	–
comRAT-G correct	–	0.48^***^	–	0.65^***^
B-JB	0.56^***^	–	–	–
B-JB correct	–	0.38^***^	–	–
Linguistic RAT	0.70^***^	–	–	–
Linguistic RAT correct	–	0.47^***^	–	–

Correlations significance level indicated as follows: 0.05 – “*”; 0.01 – “**”; 0.001 – “

*** *” N = 170*.

The correlations for the individual samples of F8 and MTurk participants can be found in the Supporting Information section at the end. No significant correlations were found between the self-ratings of creativity or problem solving skills and their performance in vRAT or linguistic RAT.

#### 4.2.3. Validity

For checking the reliability of the data, Cronbach's alpha was calculated with all RAT accuracy metrics of all the samples as shown in Table 6. The Cronbach's alpha for response time metrics is shown in Table 7. All internal validity results for the visual RAT are above 0.75 showing that the results have high internal consistency.

**Table 6:** Cronbach's alphas of the accuracy metrics of Study-2.

	**vRAT**	**ling RAT**
F8	0.81	0.94
Mturk	0.82	0.81
Combined	0.82	0.85

**Table 7:** Cronbach's alphas of the response time metrics of Study-2.

	**vRAT**	**ling RAT**
F8 all	0.93	0.91
F8 correct	0.77	0.91
MTurk all	0.83	0.87
MTurk correct	0.82	0.89
Combined all	0.85	0.90
Combined correct	0.81	0.90

## 5. Discussion

This paper focused on the creation and validation of a set of visual RAT queries. As can be seen from the results, the validation of the visual queries was successful. A significant and positive correlation has been seen between the performance of the visual RAT queries solvers, and their previous performance in linguistic queries given in a previous session (Study 1) or in the same session (Study 2). This shows that the visual RAT queries may be a way to capture the associative factor of creativity in the visual domain, and that future versions of the RAT could be given, using these stimuli, in two sensory modalities.

A few participants presented an interesting example of performing very well on one of the RATs and very poorly or averagely on the other. For example, participant 42802 from Study-2 performed exceptionally well in vRAT queries (*vRAT accuracy* = 37, vRAT mean = 15.62) but had an average *linguistic RAT accuracy* (=24, linguistic RAT mean = 20.71). In Study-2, participant 69355 had an extraordinary *linguistic RAT accuracy* (=45, linguistic RAT mean = 20.71) but only had an above average performance in vRAT (*vRAT accuracy* = 20, vRAT mean = 15.62). Participant 28941 from Study-2 performed very well in both vRAT (*vRAT accuracy* = 25) and linguistic RAT queries (*linguistic RAT accuracy* = 33). In Study-1, participant 97828 had a very high *vRAT accuracy* of 38 (vRAT mean = 20.94) but had an average *linguistic RAT accuracy* of 54 (linguistic RAT mean = 51.26) in our previous linguistic RAT study from which they were recruited. These few outlier cases open the door to interesting questions regarding how much such outliers rely on visual versus linguistic skill (rather than some pure form of association skill), or have their association skill in one modal domain much stronger than the one in the other domain. Whether and how association skill can be analyzed separately from modal ability is a question for further work.

One of the advantages of being able to give the RAT in a different modality is that such a version can be administered to participants with different native languages. In parallel to this work, the set of visual RAT queries created here was given by other authors to Finnish and Russian speakers (Toivainen et al., 2019); a correlation was observed between performance in solving the linguistic RAT in each of those languages and the visual RAT [Russian: *r*(65)=0.56, *p* < 0.001, *n* = 67 and Finnish: *r*(65) = 0.28, *p* = 0.02, *n* = 67]. A high Cronbach alpha was maintained across the different language populations (Russian: vRAT α = 0.79, lingvRAT α = 0.83; Finnish: vRAT α = 0.84, lingvRAT α = 0.73).

These empirical studies show that the visual RAT is a highly reliable tool, related to the linguistic RAT. What they do not yet show is to what extent the queries are processed visually. Future experimentation with fMRI or EEG equipment would be necessary to make any statements on this matter. The contribution or collaboration of any neuroscientists in this direction is most welcomed.

As pointed by our reviewers, to which extent linguistic associations are used by participants solving the visual RAT and visual associations when solving the linguistic RAT is hard to currently assess. One way to do this would be to establish a set of queries which have both linguistic and visual associations, and observe which solving route participants are most likely to take (and whether this is dependent on their skill in those particular domains).

As future work, we plan to implement the mechanisms for comRAT-C (Olteţeanu and Falomir, 2015) in a computational solver for the visual Remote Associates Test. This will allow us to check whether the correlation between the comRAT-C probability and human performance in linguistic queries (*r* = 0.49, *p* < 0.002) is maintained in the visual domain between a computational solver and human performance. A computational implementation of a visual RAT solver would require the gathering of data on association strength in the visual domain.

A very interesting potential future step would be the application of the RAT formalization to create RAT stimuli in a third modality. Currently, auditory and smell modalities are considered as possibilities. The initial difficulties encountered with the smell modality are related to the stimuli themselves. Attempting to obtain the stimuli from perfumers and consulting with them on the matter has made us aware that part of them are reluctant to describe their craft as a representational art (that is have smell indicate or stand in for objects), but rather as a trigger for other sensations and emotions (e.g., various smell combinations standing in for “freshness”). Thus artists of the smell modality already operate, to a certain extent, with associations. Except these associations may not point to a distinct object, but to a quality or sensation which may be, at times, hard to describe linguistically.

If comRAT-V could solve the visual RAT, it would be interesting from a computational perspective to obtain a secondary measurement for the associative process in CreaCogs, in a form of a different task. Our initial work in the direction of a second task is the Codenames board game (Zunjani and Olteteanu, 2019).

In summary, a good size set of visual RAT stimuli has been proposed and validated as part of this paper. The results show our approach to creating a visual RAT is successful. The visual stimuli will be made available to other researchers for further validation, and for scholarly pursuit of a deeper understanding of the associative factor in creativity. This work opens the path to multi-modal exploration of the association creativity process.

## Data Availability Statement

The datasets generated for this study are available on request to the corresponding author.

## Ethics Statement

Ethical review and approval was not required for the study on human participants in accordance with the local legislation and institutional requirements. The patients/participants provided their written informed consent to participate in this study.

## Author Contributions

A-MO conceptualized the visual RAT and designed the experiments for its validation. FZ contributed in the programming and deployment of the experiments online, and did the ratings and the analysis of the data collected from the experiments (except the first study). All authors wrote sections of the manuscript, revision, read, and approved the submitted version.

### Conflict of Interest

The authors declare that the research was conducted in the absence of any commercial or financial relationships that could be construed as a potential conflict of interest.

## Footnotes

^1^Figure-Eight (formerly known as CrowdFlower) is a popular crowd-sourcing platform (http://figure-eight.com).

^2^jsPsych is a JavaScript library for running behavioral experiments in a web browser (https://jspsych.org).

^3^Amazon Mechanical Turk is another popular crowd-sourcing platform (https://www.mturk.com).

## References

[B1] Ansburg P. I. (2000). Individual differences in problem solving via insight. Curr. Psychol. 19, 143–146. 10.1007/s12144-000-1011-y

[B2] Ansburg P. I. Hill K. (2003). Creative and analytic thinkers differ in their use of attentional resources. Personal. Indiv. Diff. 34, 1141–1152. 10.1016/S0191-8869(02)00104-6

[B3] Bowden E. M. Jung-Beeman M. (2003). Normative data for 144 compound remote associate problems. Behav. Res. Methods 35, 634–639. 10.3758/BF03195543 14748508

[B4] Cai D. J. Mednick S. A. Harrison E. M. Kanady J. C. Mednick S. C. (2009). Rem, not incubation, improves creativity by priming associative networks. Proc. Natl. Acad. Sci. U.S.A. 106, 10130–10134. 10.1073/pnas.0900271106 19506253 PMC2700890

[B5] Cunningham J. B. MacGregor J. Gibb J. Haar J. (2009). Categories of insight and their correlates: An exploration of relationships among classic-type insight problems, rebus puzzles, remote associates and esoteric analogies. J. Creat. Behav. 43, 262–280. 10.1002/j.2162-6057.2009.tb01318.x

[B6] Dorfman J. Shames V. A. Kihlstrom J. F. (1996). Intuition, incubation, and insight: implicit cognition in problem solving, in Implicit Cognition, ed Underwood G. (Oxford: Oxford University Press), 257–296.

[B7] Kim K. H. (2006). Can we trust creativity tests? A review of the Torrance Tests of Creative Thinking (TTCT). Creat. Res. J. 18, 3–14. 10.1207/s15326934crj1801-2

[B8] Landmann N. Kuhn M. Piosczyk H. Feige B. Riemann D. Nissen C. (2014). Entwicklung von 130 deutsch sprachigen Compound Remote Associate (CRA)-Wortraetseln zur Untersuchung kreativer Prozesse im deutschen Sprachraum. Psychol. Rundschau 65, 200–211. 10.1026/0033-3042/a000223

[B9] Mednick S. A. Mednick M. (1971). Remote Associates Test: Examiner's Manual. Boston, MA: Houghton Mifflin.

[B10] Olteţeanu A.-M. (2014). Two general classes in creative problem-solving? An account based on the cognitive processes involved in the problem structure - representation structure relationship, in Proceedings of the International Conference on Computational Creativity, eds Besold T. Kühnberger K.-U. Schorlemmer M. Smaill A. (Osnabrück: Publications of the Institute of Cognitive Science).

[B11] Olteţeanu A.-M. (2016). From simple machines to Eureka in four not-so-easy steps. Towards creative visuospatial intelligence, in Philosophy and Theory of Artificial Intelligence, Vol. 376, ed Müller V. (Berlin: Springer; Synthese Library), 159–180. 10.1007/978-3-319-26485-1_11

[B12] Olteţeanu A.-M. Falomir Z. (2015). comRAT-C: a computational compound remote associate test solver based on language data and its comparison to human performance. Patt. Recogn. Lett. 67, 81–90. 10.1016/j.patrec.2015.05.015

[B13] Olteţeanu A.-M. Schöttner M. Schuberth S. (2019a). Computationally resurrecting the functional remote associates test using cognitive word associates and principles from a computational solver. Knowl-Based Syst. 168, 1–9. 10.1016/j.knosys.2018.12.023

[B14] Olteţeanu A.-M. Schultheis H. (2017). What determines creative association? revealing two factors which separately influence the creative process when solving the remote associates test. J. Creat. Behav. 53, 389–395. 10.1002/jocb.177

[B15] Olteţeanu A.-M. Schultheis H. Dyer J. B. (2017). Computationally constructing a repository of compound Remote Associates Test items in American English with comRAT-G. Behav. Res. Methods Instrum. Comput. 50, 1971–1980. 10.3758/s13428-017-0965-8 29235071

[B16] Olteţeanu A.-M. Taranu M. Ionescu T. (2019b). Normative data for 111 compound remote associates test problems in Romanian. Front. Psychol. 10:1859. 10.3389/fpsyg.2019.01859 31551842 PMC6737282

[B17] Patterson J. (2011). F-A-S Test. New York, NY: Springer.

[B18] Salvi C. Costantini G. Bricolo E. Perugini M. Beeman M. (2016). Validation of Italian rebus puzzles and compound remote associate problems. Behav. Res. Methods 48, 664–685. 10.3758/s13428-015-0597-9 26148823

[B19] Shen W. Yuan Y. Liu C. Yi B. Dou K. (2016). The development and validity of a chinese version of the compound remote associates test. Am. J. Psychol. 129, 245–258. 10.5406/amerjpsyc.129.3.0245 29558590

[B20] Toivainen T. Olteteanu A.-M. Repeykova V. Likhanov M. Kovas Y. (2019). Visual and linguistic stimuli in the remote associates test: A cross-cultural investigation. Front. Psychol. 10:926. 10.3389/fpsyg.2019.00926 31105627 PMC6498948

[B21] Ward J. Thompson-Lake D. Ely R. Kaminski F. (2008). Synaesthesia, creativity and art: what is the link? Br. J. Psychol. 99, 127–141. 10.1348/000712607X204164 17535472

[B22] Worthen B. R. Clark P. M. (1971). Toward an improved measure of remote associational ability. J. Educ. Measure. 8, 113–123.

[B23] Wu C.-L. Chen H.-C. (2017). Normative data for chinese compound remote associate problems. Behav. Res. Methods 49, 2163–2172. 10.3758/s13428-016-0849-3 28342070

[B24] Zunjani F. H. Olteteanu A.-M. (2019). Towards reframing codenames for computational modelling and creativity support using associative creativity principles, in Proceedings of the 2019 on Creativity and Cognition, C&C '19 (New York, NY: ACM), 407–413. 10.1145/3325480.3325510

