# Supplementary material for: A Visual Remote Associates Test and Its Validation
Source: Front Psychol. 2020 Jan 28;11:26. doi: 10.3389/fpsyg.2020.00026 (PMC6997336; doi:10.3389/fpsyg.2020.00026)
Supplement: Supplementary file 1 [file Data_Sheet_1.pdf]

Supporting Information

S1 Table. Correlations of vRAT score with all the other metrics for F8 participants. Correlations significance level indicated as follows: 0.05 — '*'; 0.01 — '**'; 0.001 – '***'. N = 26.

	F	A	S	FAS	Category	vRAT	comRAT-G	B-JB	linguistic RAT
**F**	-	0.83***	0.81***	-	0.55**	0.00	0.25	0.11	0.18
**A**		-	0.86***	-	0.72***	0.16	0.54**	0.32	0.46*
**S**			-	-	0.77***	0.17	0.51**	0.30	0.43*
**FAS**				-	0.73***	0.12	0.47*	0.26	0.39*
**Category**					-	0.17	0.62***	0.29	0.47*
**vRAT**						-	0.45*	0.39*	0.47*
**comRAT-G**							-	0.57**	-
**B-JB**								-	-
**linguistic RAT**									-

S2 Table. Correlations of vRAT score with all the other metrics for MTurk participants. Correlations significance level indicated as follows: 0.05 — '*'; 0.01 — '**'; 0.001 – '***'. N = 144.

	F	A	S	FAS	Category	vRAT	comRAT-G	B-JB	linguistic RAT
**F**	-	0.68***	0.70***	-	0.54***	0.03	0.25**	0.20*	0.26**
**A**		-	0.75***	-	0.56***	0.01	0.23**	0.11	0.19*
**S**			-	-	0.60***	0.01	0.20*	0.21*	0.25**
**FAS**				-	0.63***	0.02	0.25**	0.20*	0.26**
**Category**					-	-0.03	0.20*	0.25**	0.28***
**vRAT**						-	0.31***	0.17*	0.27**
**comRAT-G**							-	0.29***	-
**B-JB**								-	-
**linguistic RAT**									-

S3 Table. Correlations of response times for the metrics for the F8 participants of Study-2. Correlations significance level indicated as follows: 0.05 — '*'; 0.01 — '**'; 0.001 – '***'. N = 26.

	vRAT	vRAT correct	B-JB	B-JB correct
**comRAT-G**	0.87***	-	0.23	-
**comRAT-G correct**	-	0.51**	-	0.48*
**B-JB**	0.54**	-	-	-
**B-JB correct**	-	0.70***	-	-
**linguistic RAT**	0.93***	-	-	-
**linguistic RAT correct**	-	0.76***	-	-

S4 Table. Correlations of response times for the metrics for the MTurk participants of Study-2. Correlations significance level indicated as follows: 0.05 — '*'; 0.01 — '**'; 0.001 – '***'. N = 144.

	**vRAT**	**vRAT correct**	**B-JB**	**B-JB correct**
**comRAT-G**	0.61***	-	0.68***	-
**comRAT-G correct**	-	0.54***	-	0.66***
**B-JB**	0.58***	-	-	-
**B-JB correct**	-	0.43***	-	-
**linguistic RAT**	0.65***	-	-	-
**linguistic RAT correct**	-	0.50***	-	-
